# A Temperature-Robust Envelope Detector Receiving OOK-Modulated Signals for Low-Power Applications

**DOI:** 10.3390/s24196369

**Published:** 2024-09-30

**Authors:** Alessia Maria Elgani, Matteo D’Addato, Luca Perilli, Eleonora Franchi Scarselli, Antonio Gnudi, Roberto Canegallo, Giulio Ricotti

**Affiliations:** 1 STMicroelectronics, 20864 Agrate Brianza e Cornaredo, Italy; matteo.daddato2@unibo.it (M.D.); roberto.canegallo@st.com (R.C.); giulio.ricotti@st.com (G.R.); 2 Advanced Research Center on Electronic Systems “Ercole De Castro” (ARCES), University of Bologna, 40123 Bologna, Italy; luca.perilli@unibo.it (L.P.); eleonora.franchi@unibo.it (E.F.S.); antonio.gnudi@unibo.it (A.G.); 3 Department of Electrical, Electronic and Information Engineering “Guglielmo Marconi” (DEI), University of Bologna, 40123 Bologna, Italy

**Keywords:** envelope detector, temperature compensation, ultra-low-power, wake-up receivers (WuRXs)

## Abstract

This paper presents a passive Envelope Detector (ED) to be used for reception of OOK-modulated signals, such as in Wake-Up Receivers employed within Wireless Sensor Networks, widely used in the IoT. The main goal is implementing a temperature compensation mechanism in order to keep the passive ED input resistance roughly constant over temperature, making it a constant load for the preceding matching network and ultimately keeping the overall receiving chain sensitivity constant over temperature. The proposed ED was designed using STMicroelectronics 90 nm CMOS technology to receive 1 kbps OOK-modulated packets with a 433 MHz carrier frequency and a 0.6 V supply. The use of a block featuring a Proportional-to-Absolute Temperature (PTAT) current yields a 5 dB reduction in sensitivity temperature variation across the −40 °C to 120 °C range. Moreover, two different implementations were compared, one targeting minimal mismatch and the other one targeting minimal area. The minimal area version appears to be better in terms of estimated overall chain sensitivity at all temperatures despite a higher sensitivity spread.

## 1. Introduction

Envelope Detectors (EDs) are used in several applications involving an incoming high-frequency amplitude modulated signal whose envelope needs to be extracted.

A usage example for EDs is that of Wake-Up Receivers (WuRXs), widely employed in the Internet of Things (IoT). IoT nodes are organized in subnetworks, the Wireless Sensor and Actuator Networks (WSAN), typically composed of a central node; the gateway, which manages all communication within the network; and several end nodes communicating wireless.

In such nodes, the transceiver is typically the most power-hungry section, and a popular power reduction method is the use of WuRXs. The WuRX is an always-on ultra-low-power additional receiver, which can be integrated in end nodes to continuously monitor the channel instead of the main transceiver and activate the rest of the node only when a Wake-Up packet is received from the gateway. This approach allows event-driven, asynchronous communication [1,2,3,4,5,6,7].

Despite its noise sensitivity, low-power applications mostly use OOK modulation, which is the simplest kind of Amplitude Shift Keying modulation, the digital form of Amplitude Modulation [1,8]. The modulating signal is a square wave: the carrier is present during the transmission of a logic 1, whereas there is no carrier during the transmission of a logic 0. It is frequently employed in communication, such as RF and optical communication, thanks to its simplicity and spectral efficiency.

OOK demodulation is easily carried out by leveraging the second-order non-linearities of MOSFETs in subthreshold, which is also beneficial for low-power operation. Two approaches are available [9], the active [10,11] and the passive one [12,13,14,15,16,17,18,19,20,21]. Unlike in active EDs, in passive ones, demodulating MOSFETs or off-the-shelf diodes (in the case of discrete-component solutions) are biased with zero current and the RF signal is coupled to MOSFET terminals by capacitances. No static current results in an inherent absence of flicker noise. This directly translates into an enhanced sensitivity, i.e., the minimum input power that can be correctly detected. Yet, a trade-off exists between the propagation delay and the rectification gain [1]. Then, the demodulation mechanism of passive EDs is heavily dependent on the input RF frequency due to the presence of the coupling and filtering capacitances. Finally, more constraints exist in terms of the maximum input power that can be correctly received than for active EDs (Section 3.4) [11].

For optimal communication, ED operation needs to be reliable in all settings, where environmental conditions, such as temperature, may vary widely. Therefore, it is advisable to implement some sort of compensation in order for the ED to be fully functional in every condition. This paper accomplishes this task by adding a nanowatt temperature compensation block to a passive ED for robust operation in case of significant temperature variations. Two different versions are implemented, the first targeting minimal mismatch and the second targeting minimal area, for comparison in terms of room temperature sensitivity and sensitivity spread due to mismatch.

This paper is organized as follows: Section 2 presents the architectural choices for the passive ED with the Proportional-to-Absolute Temperature (PTAT) compensation block, comparing them with other EDs in the literature. In Section 3, the passive ED is thoroughly described, from the basic structure presented in [12] to the proposed temperature compensation mechanism and the reasons and consequences for choosing a differential approach. Section 4 shows the validation of the proposed temperature compensation mechanism as well as the comparison between the two implementations. Finally, Section 5 concludes the paper.

## 2. State-of-the-Art Passive EDs with Temperature Compensation

As mentioned in the Introduction, the demodulation mechanism leverages the second-order non-linearities of MOSFETs in subthreshold [10]. The standard passive ED is a Dickson charge pump composed of diode-connected MOSFETs biased with zero static current and capacitances.

**Figure 1:**
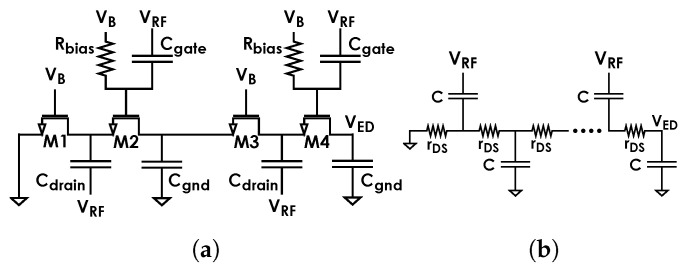
(**a**) Passive ED as in [12] and (**b**) estimation of its propagation delay.

**Figure 2:**
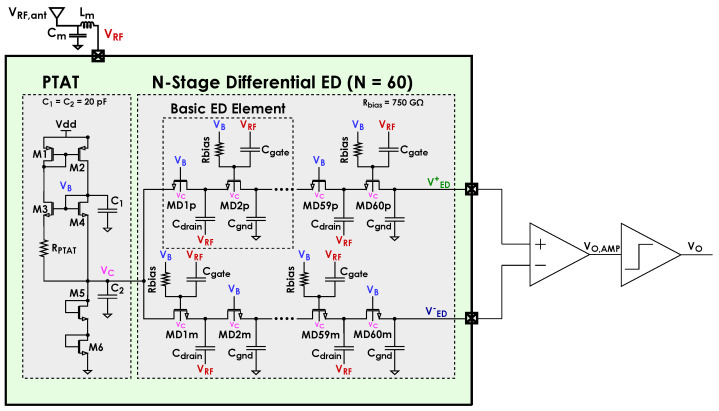
Proposed ED with temperature compensation [22].

The proposed ED employs the variation presented in [12] and shown in Figure 1a, where voltage $V_{B}$ is generated by an additional PTAT block as in Figure 2. The impedance seen at the RF input is the parallel connection between a resistance $R_{in}$ and a capacitance $C_{in}$ and is matched to the antenna impedance by a matching network. The temperature compensation mechanism operates through a dedicated block aiming at temperature dependence reduction for $R_{in}$ so that the temperature dependence of matching network gain $A_{v}$ is also minimized. As detailed in Section 3.2, this ultimately reduces the temperature dependence of the overall receiving chain sensitivity.

Temperature compensation techniques have already been presented. An alternative approach employing a different additional block was proposed in [15]. It requires both enhancement and depletion MOSFETs as it leverages the threshold difference between them and demonstrates a reduction in sensitivity temperature dependence over a −10 °C to 50 °C operating temperature range.

Moreover, a structure similar to the one proposed in this paper, employing a PTAT current for the biasing of self-mixer gate terminals, was proposed in [16]. Yet, a discussion of sensitivity dependence over temperature is missing.

In [17], a dedicated block is used to drive diode bulk terminals so as to make their threshold voltage, thus their channel resistance, constant over temperature with the ultimate aim of a constant ED bandwidth. The operating temperature range is −10 °C to 40 °C.

Ref. [18] targets operating temperatures between −30 °C and 70 °C. It focuses on temperature and supply robust voltage and current references, as well as comparator threshold adjustments by means of a capacitive digital-to-analog converter for temperature compensation.

Finally, ref. [19] relies on a charge-transfer summation amplifier following the ED for temperature robustness across a −40 °C to 85 °C operating temperature range.

Table 1 compares the different implementations addressing sensitivity robustness through a Figure of Merit, which represents the inverse of the mean sensitivity variation across 1 °C $\left((T_{max}-T_{min}\right)/\Delta P_{sens}$). As a result, our implementation shows FoM performances aligned with the state-of-the-art [18] over a wider temperature range.

## 3. ED and PTAT Block Description

### 3.1. ED Model and Receiving Chain Sensitivity Optimization

For the sake of clarity, this subsection summarizes the features of the employed ED architecture just as in [12]. Its topology (Figure 1a) is a variation of the Dickson charge pump. The following is based on the standard subthreshold current model for the MOSFET-based diodes:

(1) $$I_{DS}=I_{S}\frac{W}{L}e^{\frac{V_{GS}}{nV_{T}}}\left(1-e^{\frac{-V_{DS}}{V_{T}}}\right),$$

where

(2) $$I_{S}=I_{S0}e^{\frac{-V_{TH}}{nV_{T}},}$$

W/L is the aspect ratio of the MOSFET and $I_{S0}$ is a technological parameter. Assuming an input OOK-modulated signal $V_{RF,ant}\left(t\right)=V_{M}\left(t\right)\cos\left(\omega t\right)$, where $V_{M}\left(t\right)$ is the envelope signal and is equal to $V_{M0}$ when a ‘1’ is transmitted and equal to 0 when a ‘0’ is transmitted, the output amplitude is [12]

(3) $$V_{ED}=\frac{NV_{M0}^{2}}{4nV_{T}},$$

where *N* is the number of diode stages, *n* is the non-ideality factor of the diodes and $V_{T}$ is the thermal voltage.

**Figure 3:**
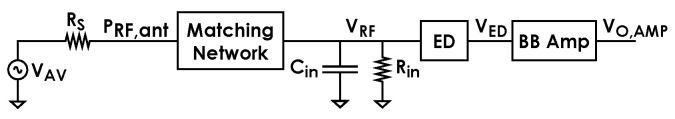
The typical WuRX chain excluding the final digitizing element, as in [12].

Figure 3 shows the typical receiving chain of an ultra-low-power system, such as that of a WuRX, excluding the final digitizing element. It is composed of a matching network, ED and baseband amplifier. As mentioned, at RF, the ED can be seen as a load composed of a resistance and a capacitance in parallel, $R_{in}$ and $C_{in}$, for the preceding matching network. This implies that the matching network is designed based on $R_{in}$ and $C_{in}$ values. The values for $C_{gate}$, $C_{drain}$ and $C_{gnd}$ of Figure 2 are mainly determined by the RF frequency they need to correctly transfer to the internal diode nodes and filter out, respectively. Assuming $C_{gate}$, $C_{drain}$ and $C_{gnd}$ to be big enough with respect to the $C_{gs}$ of the diodes and to have negligible parasitics, $R_{in}=r_{DS}/N$, where $r_{DS}$ is the channel resistance of diodes, and $C_{in}=C_{in}^{\prime }+C_{pad}=NC_{gs}+C_{pad}$, where $C_{pad}$ is the parasitic capacitance of the RF input pad.

The delay introduced by the ED and, thus, its maximum operating bitrate, can be found by applying the open circuit time constant method to the equivalent circuit in Figure 1b, assuming all capacitances to have the same value. This yields [20]

(4) $$\tau ∼Cr_{DS}\frac{N\left(N+1\right)}{2}=CR_{in}\frac{N^{2}\left(N+1\right)}{2}.$$

Therefore, τ is almost proportional to the cube of *N*, which results in any increase in *N* having a strong impact on the delay introduced by the circuit.

As mentioned, Figure 3 shows the typical receiving chain of an ultra-low-power system excluding the final digitizing element. This is useful to estimate the overall chain sensitivity [12]. The following analysis is carried out assuming the matching condition to be met. First, the input power is $P_{RF,ant}=V_{RF,ant}^{2}/2R_{S}$, where $R_{S}=50\;\Omega$ is the resistance of the source. This implies the voltage amplitude virtually needed within the generator before $R_{S}$ is $V_{AV}=2V_{RF,ant}$. The peak-to-peak signal at the output of the matching network is then $V_{RF}=A_{v}\;V_{RF,ant}$, with $A_{v}$ being the matching network gain. Since it is the most practical implementation for the matching network, a commercial component simple LC network such as the one shown in Figure 2 shall be considered in the following, yielding [12]

(5) $$A_{v}=\sqrt{\frac{R_{in}}{R_{S}}}/\sqrt{\left(1+\frac{\omega R_{in}C_{in}}{Q_{ind}}\right)},$$

where $Q_{ind}$ is the Q factor of the matching network inductance. Necessary conditions for high $A_{v}$ are low $C_{in}$ and high $Q_{ind}$ at the target RF carrier frequency.

The peak-to-peak signal at the output of the ED is $V_{ED}=\left(NA_{v}^{2}P_{RF,ant}2R_{S}\right)/\left(4nV_{T}\right)$ from (3). Since the diodes have no flicker noise, the noise power at the output of the ED is $V_{N,ED}^{2}\left(f\right)f_{N}=4k_{B}TNr_{DS}f_{N}=4k_{B}TN^{2}R_{in}f_{N}$, where $k_{B}$ is the Boltzmann constant and $f_{N}$ is the ED noise bandwidth, which is ideally equal to bitrate $f_{S}$. Then, the SNR at the ED output is

(6) $$SNR_{ED,o}=\frac{V_{ED}^{2}}{V_{N,ED}^{2}\left(f\right)f_{N}}=\frac{A_{v}^{4}P_{RF,ant}^{2}R_{S}^{2}}{{\left(4nV_{T}\right)}^{2}k_{B}TR_{in}f_{N}}.$$

Thus, the SNR ultimately does not depend on *N*.

The ED is followed by a baseband amplifier with Noise Factor NF, which can be expressed as

(7) $$NF=1+\frac{N_{amp,o}}{\int G{\left(f\right)}^{2}V_{N,ED}^{2}\left(f\right)df},$$

where $N_{amp,o}$ is the integrated noise at the amplifier output due to the amplifier itself, *G* is the amplifier voltage gain and $V_{N,ED}^{2}$ is as defined previously. Assuming $SNR_{req}$ to be the minimum required SNR at the output of the baseband amplifier, the overall chain sensitivity can be calculated as

(8) $$P_{SEN}=\sqrt{\frac{SNR_{req}NF{\left(4nV_{T}\right)}^{2}k_{B}TR_{in}f_{N}}{A_{v}^{4}R_{S}^{2}}},$$

where $A_{v}$ depends on $R_{in}$ through (5).

This expression as a function of $R_{in}$ has a minimum [12], and the optimum $\bar{R_{in}}$ must be chosen accordingly in order to optimize chain sensitivity. As mentioned, $SNR_{ED,o}$ in (6) does not depend on *N*, whereas a big value for *N* is desirable for a small NF (7), as NF directly degrades sensitivity (8). This poses a trade off between sensitivity and bitrate, which is closely linked to the propagation delay the ED introduces τ, as in (4). Moreover, a big value for *N* also results in a high $C_{in}$, thus degrading $A_{v}$. Therefore, *N* becomes an important design parameter in a complicated trade-off, as it affects NF, $A_{v}$, $R_{in}$ and τ [1].

This leads to the choice of an optimum $r_{DS}$, $\bar{r_{DS}}=N\bar{R_{in}}$. Thanks to the possibility of setting the $V_{GS}$ of the diodes, $\bar{r_{DS}}$ can be found through the following formula:

(9) $$\bar{r_{DS}}=\frac{1}{\bar{g_{DS}}}={\left[\frac{\partial i_{DS}}{\partial v_{DS}}\right]}^{-1}≃\frac{V_{T}}{I_{S}{\left(\frac{W}{L}\right)}_{MD}}e^{-\frac{\bar{V_{GS}}}{nV_{T}}},$$

given that $V_{DS}∼0$ [12] and where MD are the diodes.

Ultimately, by setting the appropriate diode $\bar{V_{GS}}$, it is possible to set the correct $R_{in}$ for chain sensitivity optimization. All useful formulas for sensitivity optimization are reported in Table 2.

### 3.2. Temperature Compensation through the PTAT Block

Actually, $P_{SEN}$ as in (8) depends on temperature through $f_{N}$ as well, which in turn is inversely proportional to the baseband output resistance $R_{out}=Nr_{DS}=N^{2}R_{in}$. Therefore, the integrated noise at the output of the ED becomes

(10) $$\int V_{N,ED}^{2}\left(f\right)df\propto \frac{4k_{B}TN^{2}R_{in}}{N^{2}R_{in}}=4k_{B}T,$$

which is independent of $R_{in}$.

As for NF of the baseband amplifier following the ED, we shall assume the amplifier to be standard, for instance a common-source with an active load, with negligible residual flicker noise and transconductance $g_{m}$, for the sake of simplicity, and to operate in the subthreshold region. Let us also assume the baseband amplifier to have a bandwidth close to bitrate $f_{S}$, which is in turn close to the ED noise bandwidth $f_{N}$. In this case, (7) results in

(11) $$NF_{SE}=1+\frac{2\times \frac{4k_{B}T}{g_{m}}{\left(g_{m}R_{out}\right)}^{2}f_{S}}{{\left(g_{m}R_{out}\right)}^{2}\times 4k_{B}TN^{2}R_{in}f_{N}}=1+\frac{2}{g_{m}N^{2}R_{in}},$$

If *N* or $g_{m}$ are big enough, e.g., a 3.3−μS $g_{m}$ is enough for a $2.2-k\Omega \;R_{in}$ and N=60 (see Section 4.2.1), NF is close enough to unity at room temperature and its variations with temperature are negligible.

This results in $A_{v}$, as in (5), being the only element with a dependence on $R_{in}$ in (8). In turn, $R_{in}$ is a very rapidly varying function of temperature *T* through $r_{DS}$ as in (9), which ultimately results in

(12) $$P_{SEN}\propto \sqrt{\frac{T^{3}}{{\left(A_{v}\left(R_{in}\left(T\right)\right)\right)}^{4}}}.$$

The purpose of the proposed compensation mechanism is to make $R_{in}$, and thus $A_{v}$, constant with temperature leaving $P_{SEN}\propto \sqrt{T^{3}}$. No further attempt has been made to cancel this residual temperature dependence. An additional effect is making $f_{N}$ constant as well.

The following analysis is carried out assuming a small input signal, i.e., close to sensitivity, resulting in source voltages roughly equal to $V_{C}$ for all diodes. As illustrated in Figure 2, the PTAT block generates a $V_{GS}$ for the diodes so as to make their $r_{DS}$, and thus $R_{in}$, roughly constant with *T*. This also results in a roughly constant $A_{v}$ so that no changes to the matching network are required in case of temperature changes. The compensation mechanism is again based on the standard subthreshold current model for the diodes (1), (2). The PTAT provides the diodes with the same $V_{GS}$ as M4, i.e., $V_{GS}=V_{GS,M4}=V_{B}-V_{C}$. Assuming the diodes to have a small $V_{DS}$ and assuming the difference in $V_{DS}$ between the diodes and M4 not to significantly affect $V_{TH}$,

(13) $$r_{DS}=\frac{V_{T}}{I_{S}{\left(\frac{W}{L}\right)}_{MD}}e^{\frac{-V_{GS}}{nV_{T}}}=\frac{V_{T}}{I_{S}{\left(\frac{W}{L}\right)}_{MD}}e^{\frac{-V_{GS,M4}}{nV_{T}}}.$$

From (1), assuming $V_{DS,M4}⩾3V_{T}$, the bias current of M4 is

(14) $$I_{M4}=I_{S}{\left(\frac{W}{L}\right)}_{M4}e^{\frac{V_{GS,M4}}{nV_{T}}}.$$

Substituting (14) in (13), and again assuming the difference in $V_{DS}$ between the diodes and M4 not to significantly affect $V_{TH}$,

(15) $$r_{DS}=\frac{V_{T}}{I_{M4}}\frac{{\left(\frac{W}{L}\right)}_{M4}}{{\left(\frac{W}{L}\right)}_{MD}}.$$

Due to the 1:1 mirror M1–M2, the bias current of M4 is equal to that of M3 and is

(16) $$I_{M4}=I_{M3}=\frac{nV_{T}}{R_{PTAT}}\ln\frac{{\left(\frac{W}{L}\right)}_{M3}}{{\left(\frac{W}{L}\right)}_{M4}}.$$

Therefore, combining (16) with (15), it is possible to prove that

(17) $$R_{in}=\frac{r_{DS}}{N}=\frac{1}{N}\frac{{\left(\frac{W}{L}\right)}_{M4}}{{\left(\frac{W}{L}\right)}_{MD}}\frac{R_{PTAT}}{n\ln\frac{{\left(W/L\right)}_{M3}}{{\left(W/L\right)}_{M4}}},$$

which shows that $R_{in}$ is theoretically not a function of *T*. A simple start-up circuit, shown in Figure 2 for the sake of simplicity, is also included to push the PTAT block away from its zero-current stable DC operating point and push it to the actual DC operating point it was designed for.

### 3.3. ED Differential Approach

**Figure 4:**
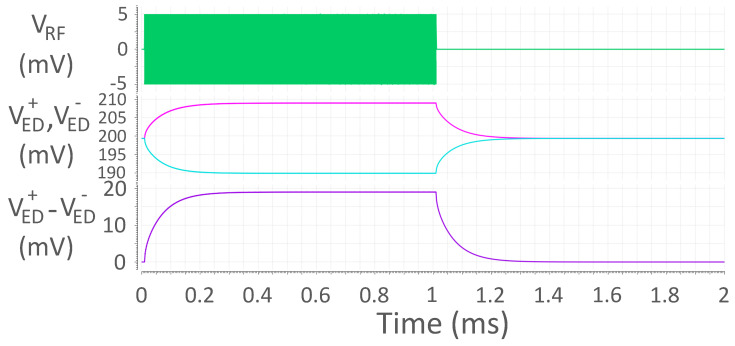
Sample simulated waveforms in a differential passive ED for $V_{M}$ = 5 mV. From top to bottom: ED input signal $V_{RF}$, ED single-ended outputs $V_{ED}^{+}$ and $V_{ED}^{-}$, and ED differential output $V_{ED}^{+}-V_{ED}^{-}$.

The addition of the PTAT block causes its own noise, which includes both thermal and flicker—unlike that of the ED—to propagate through the signal chain. In order to prevent a heavy degradation in the achievable chain sensitivity, a differential approach was chosen: the implemented ED features two identical chains, each of which has *N* diodes, connected to the PTAT cell instead of one, as shown in Figure 2. The noise due to the PTAT cell is then seen as common-mode at the output of the ED and, thus, gets canceled out by a subsequent ideal differential amplifier. The differential circuit has an output signal

(18) $$V_{ED}=\frac{2NV_{M0}^{2}}{4nV_{T}},$$

an input resistance $R_{in}=r_{DS}/2N$ and an input capacitance $C_{in}=2NC_{gs}+C_{pad}$, which yields the same SNR at the ED output as in the single-ended case. Sample simulated waveforms are shown in Figure 4. The envelope of the incoming OOK-modulated signal is extracted with both polarities, $V_{ED}^{+}$ by the positive diode chain and $V_{ED}^{-}$ by the negative diode chain. The output is then read differentially, $V_{ED}^{+}-V_{ED}^{-}$.

Moreover, switching to a differential approach causes no increases in power consumption due to the zero-current biasing of the diodes. Since the two chains work independently, the system does not get slowed down either.

Finally, it shall be proven that the noise factor NF of the amplifier is the same when adopting a differential approach as it is in the single-ended case. The same assumptions as those in Section 3.2—a standard baseband amplifier, for instance, a common-source with an active load, operating in the subthreshold region with negligible residual flicker noise and transconductance $g_{m}$—are supposed to hold. In the differential case and assuming the comparison to be carried out at total current consumption parity, (7) results in

(19) $$NF_{diff}=1+\frac{4\times \frac{4k_{B}T}{g_{m}/2}{\left(\frac{g_{m}R_{out}}{2}\right)}^{2}f_{S}}{{\left(\frac{g_{m}R_{out}}{2}\right)}^{2}\times 4k_{B}T{\left(2N\right)}^{2}R_{in}f_{N}}=1+\frac{2}{g_{m}N^{2}R_{in}},$$

which is the same as in the single-ended case (11).

This shows that no degradation occurs in the overall SNR at the amplifier output by adopting the differential approach.

### 3.4. Maximum Input Power

**Figure 5:**
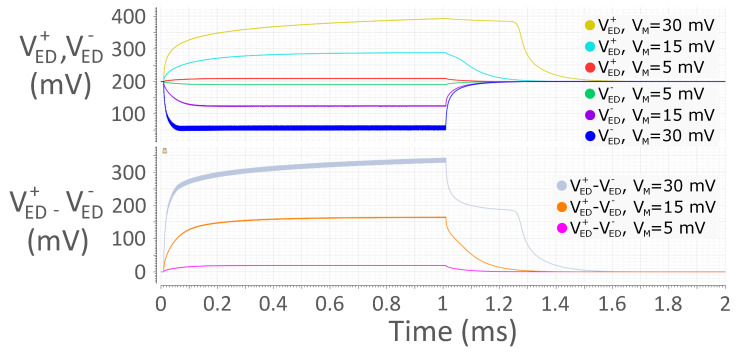
Sample simulated waveforms in a differential passive ED for $V_{M}$ = 5 mV, 15 mV and 30 mV. From top to bottom: ED single-ended outputs $V_{ED}^{+}$ and $V_{ED}^{-}$, ED differential output $V_{ED}^{+}-V_{ED}^{-}$. The picture shows ED output distortion due to increased input power.

A major drawback of passive EDs is the existence of a typically low maximum input power. This occurs both in single-ended structures employing a positive chain, as $V_{ED}^{+}$ in Figure 5 shows, and in differential structures, as proven by $V_{ED}^{+}-V_{ED}^{-}$. This Figure portrays $V_{ED}^{+}$ for $V_{M}$ = 5 mV, 15 mV and 30 mV in red, light blue and yellow, respectively, and $V_{ED}^{-}$ for $V_{M}$ = 5 mV, 15 mV and 30 mV in green, violet and blue, respectively, on top; further, it displays differential output $V_{ED}^{+}-V_{ED}^{-}$ for $V_{M}$ = 5 mV, 15 mV and 30 mV in pink, orange and gray, respectively, at the bottom. The yellow trace on top, i.e., $V_{ED}^{+}$ for $V_{M}$ = 30 mV, demonstrates that the shape of $V_{ED}^{+}$ features a delayed transition to ‘0’ when the input power is too high. This issue extends to the differential output signal $V_{ED}^{+}-V_{ED}^{-}$, as depicted by the gray trace at the bottom of the same Figure, as well as to the amplifier and comparator outputs. This is due to the body effect affecting diodes.

Let us consider the positive diode chain in Figure 2. All diodes have their body terminal connected to $V_{C}$; however, when a ‘1’ is being received, their source voltage becomes farther and farther from $V_{C}$, moving along the chain towards the output. This implies a more and more positive $V_{SB}$ is applied to the diodes moving along the chain, resulting in a higher and higher threshold voltage $V_{TH}$. Actually, the higher the input power, the stronger is this effect. At a certain point, the capacitance discharge occurring at the end of the reception of the ‘1’ becomes so difficult that it takes a non-negligible percentage of the bit-time to complete, resulting in output distortion.

If the differential approach is taken, this effect also concerns the negative diode chain in the opposite way. As a matter of fact, a more and more negative $V_{SB}$ is applied to the diodes moving along the chain, resulting in a lower and lower threshold voltage $V_{TH}$. When the reception of the ‘1’ ends, capacitance discharge on this side becomes very fast, contributing to differential signal distortion.

Possible countermeasures include connecting diode source terminals to their bulk terminals and reducing the number of stages on the fly when the input power is high. The former solution may be very detrimental to sensitivity as it would cause a capacitive divider between $C_{drain}$ and bulk parasitics in diodes having the RF signal applied to their source. The latter may be risky as well, since the switches to perform the operation may be critical in terms of capacitance, leakage or noise.

## 4. Implementation Criteria

### 4.1. Parameter Optimization

Detailed study of this ED architecture has shown there are two different sources for loss of signal within the ED with respect to the theoretical value for $V_{ED}$ (3). The first one is MOSFET junction leakage within the diodes, whereas the second one is non-perfect coupling of the input signal $V_{RF}$ to the internal gate and drain nodes. These phenomena directly translate into a reduction in the gain of the ED, which was modeled as a multiplication of the theoretical gain by a factor k<1 independent of *N*, yielding

(20) $$V_{ED}=\frac{kNV_{M0}^{2}}{4nV_{T}}$$

and ultimately, at room temperature,

(21) $$P_{SEN}=\sqrt{\frac{SNR_{req}NF{\left(4nV_{T}\right)}^{2}k_{B}TR_{in}f_{N}}{A_{v}^{4}k^{2}R_{S}^{2}}}.$$

The MOSFET type featuring the lower *n* available within the chosen technology has been selected in order to maximize ED output amplitude, as in (20). Also, a high resistivity resistor with low temperature coefficient is needed for $R_{PTAT}$. The same holds for $R_{bias}$ if implemented as an actual resistor.

As for capacitors, the choice of type and polarity for $C_{gate}$ and $C_{drain}$ may have a critical impact on sensitivity. Unless parasitics towards ground are negligible with respect to the nominal capacitance value or are evenly distributed between the two terminals of the capacitors, it is necessary to determine on which side it is best to leave most of the parasitics themselves—towards the RF input or the internal diode nodes. If non-negligible coupling capacitor parasitics are placed towards the RF input, a significant increase in the overall input capacitance of the ED, $C_{in}$, may occur, ultimately reducing the matching network gain (5). On the other hand, if non-negligible coupling capacitor parasitics are placed towards the internal diode nodes, capacitive dividers may result in a loss of signal at the diode terminals themselves. It is possible to determine which of the two situations is best by simulation. In both our versions, an assessment of this aspect has led us to place most of $C_{gate}$ and $C_{drain}$ parasitics, which amount to roughly one third of their nominal capacitance value, towards the RF input. Capacitor losses, in particular on $C_{gnd}$, also need to be assessed.

First, a preliminary design was carried out based on the formulas reported in Section 3.1, with an NF value close enough to unity in the whole operating temperature range and the following remarks, with reference to Figure 2:

- High L in M1 and M2 for good mirroring.
- High W in M1 and M2 for low $V_{GS}$ to make room for M3 and M4.
- $\frac{{\left(W/L\right)}_{3}}{{\left(W/L\right)}_{4}}=2$ and highest possible $R_{PTAT}$ allowed by the technology considering area occupation to minimize the static current of the PTAT block.
- Lowest possible $C_{gnd}$ for effectively cutting the chosen carrier RF frequency.
- Lowest possible $C_{gate}$ and $C_{drain}$ for effectively coupling the input signal to the internal diode nodes at the chosen carrier RF frequency.

However, mismatch between the diodes and MOSFET M4 may negatively affect temperature compensation precision. Let us assume that a difference $\Delta V_{TH}$ exists between the average threshold voltage of the diodes $V_{TH}$ and that of MOSFET M4, $V_{TH,M4}$, due to mismatch. Since mismatch is random, $\Delta V_{TH}$ can have both signs. Equation (15) then becomes

(22) $$r_{DS}=\frac{V_{T}}{I_{M4}}\frac{{\left(\frac{W}{L}\right)}_{M4}}{{\left(\frac{W}{L}\right)}_{MD}}e^{\Delta V_{TH}/nV_{T}},$$

so the difference between the actual $r_{DS}$ and the ideal one is

(23) $$\Delta r_{DS}=r_{DS}-r_{DS,ideal}=r_{DS,ideal}\left(e^{\Delta V_{TH}/nV_{T}}-1\right).$$

The error in $r_{DS}$ corresponding to a 1-σ error in $V_{TH}$ is then

(24) $$\Delta r_{DS,\sigma }=r_{DS,ideal}\left(e^{\sigma_{V_{TH}}/nV_{T}}-1\right)=r_{DS,ideal}\left(e^{a_{0}/nV_{T}\sqrt{W\times L}}-1\right),$$

where $a_{0}$ is the well-known Pelgrom coefficient [23]. Equation (24) shows that it is beneficial to increase W×L to ultimately reduce temperature compensation degradation due to $\Delta r_{DS,\sigma }$. This poses a design trade-off between $C_{gs}$, and thus $C_{in}$, and $\Delta r_{DS,\sigma }$.

Since the equations for $R_{in}=r_{DS}/2N$ and $C_{in}=2NC_{gs}+C_{pad}$ are somewhat ideal, parameter optimization has to be performed based on chain sensitivity optimization and temperature compensation afterwards. As for diode area, a minimal W×L for the diodes translates into a smaller $C_{gs}$, which requires smaller $C_{gate}$ and $C_{drain}$ values at a parity of signal coupling at diode terminals, and thus a smaller $C_{in}$. This results in a higher $A_{v}$ gain at $Q_{ind}$ parity, from (5). On the other hand, a non-minimal W×L for the diodes is required to increase temperature compensation accuracy (24), thus reducing sensitivity spread, even if pad parasitics may significantly contribute to $C_{in}$.

### 4.2. Two Implemented Versions

The proposed ED has been designed using an STMicroelectronics 90 nm CMOS technology with a 0.6 V supply voltage to receive 1 kbps OOK-modulated signals with a 433 MHz carrier frequency. Two different versions are provided in order to gain further insight into implementation criteria: version 1 with minimized effects of mismatch on temperature compensation, thus sensitivity, and version 2 with minimized $C_{in}$ and area.

#### 4.2.1. Version 1, Minimal Mismatch

This version has been designed with non-minimal diode W×L. Its calculated optimal $R_{in}$ at room temperature is roughly 10kΩ. *N* = 60 has been chosen based on the trade-off between an NF value close to unity and manageable $C_{in}$ and τ values—see Section 3.1. The theoretical $r_{DS}\;=\;1.2\;M\Omega$ for each diode stage results from the optimal $R_{in}$ and the chosen *N*. The actual value is $r_{DS}\;=\;1.1\;M\Omega$. The dimensions of the diodes are *W* = 450 nm × 2 and *L* = 450 nm. The values for the filtering and coupling capacitors are $C_{gate}=C_{drain}=C_{gnd}$ = 36 fF implemented as 1.2-V poly-on-pplus capacitors. The simulated $C_{in}^{\prime }$ is 1.68 pF. $R_{bias}$ has been implemented as a diode-connected MOSFET with a null $V_{GS}$. According to simulations, factor *k* is roughly 0.75. The dimensions of M4 are *W* = 450 nm and *L* = 450 nm, whereas M3 has the same dimensions as the diodes, $R_{PTAT}\;=\;3.5\;M\Omega$. A 100 nA biasing current in the baseband amplifier following the ED, which translates into a 3.3−μS$g_{m}$ in the chosen technology, would yield NF=1.08 in the worst case, namely, for $R_{in}\;=\;2.2\;k\Omega$, i.e., at 120 °C when the PTAT block is not used. This confirms that NF can be considered roughly equal to unity when calculating $P_{SEN}$. The PTAT block, start-up included, consumes 6 nW at 20 °C, 1.4 nW at −40 °C and 12.2 nW at 120 °C.

#### 4.2.2. Version 2, Minimal Area

This version has been designed with minimal diode W×L. Its calculated optimal $R_{in}$ at room temperature is roughly 10kΩ again with *N* = 60 diode stages, resulting in a theoretical $r_{DS}\;=\;1.2\;M\Omega$ and an actual $r_{DS}\;=\;1.1\;M\Omega$ for each diode. The dimensions of the diodes are *W* = 120 nm and *L* = 100 nm. The values for the filtering and coupling capacitors are $C_{gate}=C_{drain}=C_{gnd}$ = 13 fF implemented as 1.2-V poly-on-pplus capacitors. The simulated $C_{in}^{\prime }$ is 0.62 pF. $R_{bias}$ has been implemented as a diode-connected MOSFET with a null $V_{GS}$. According to simulations, factor *k* is roughly 0.75, around the same as for version 1 since junction losses are lower due to a lower diode area, but non-perfect coupling of the input signal $V_{RF}$ to the internal gate and drain nodes is more significant due to smaller coupling capacitors. The dimensions of M4 are *W* = 450 nm and *L* = 100 nm, whereas those of M3 are *W* = 450 nm × 2, *L* = 100 nm and $R_{PTAT}\;=\;1.4\;M\Omega$. Again, a 100 nA biasing current in the baseband amplifier following the ED is supposed in order to assume NF as roughly equal to unity in all cases. The PTAT block, start-up included, consumes 14 nW at 20 °C, 3.9 nW at −40 °C and 33.8 nW at 120 °C.

### 4.3. Temperature Compensation Validation

The aim of this section is the validation of the temperature compensation mechanism, i.e., proving its functionality and the performance benefits its use brings to the ED in terms of reduction in the chain sensitivity temperature variation. This has been carried out using the minimal mismatch version, as in Section 4.2.1.

**Figure 6:**
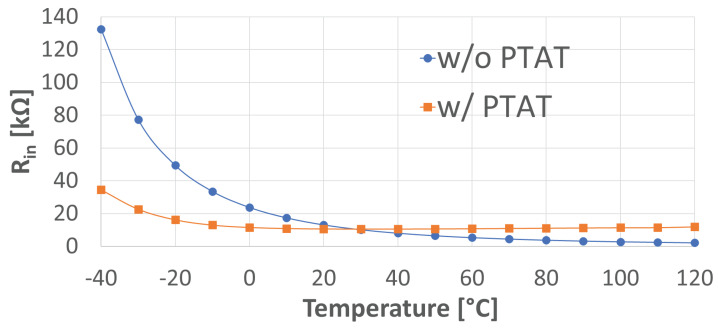
Version 1 simulated $R_{in}$ with and without the use of the PTAT block.

The temperature compensation mechanism proves to be effective in reducing the variations of $R_{in}$, which is a very rapidly varying function of temperature without the use of the PTAT, as shown in (9). Figure 6 shows the simulated $R_{in}$: variations are significantly smaller with the use of the PTAT block than without its use. It is important to note that, even with the use of the PTAT block, $R_{in}$ is not completely flat across the operating temperature range, most likely due to the fact that the assumptions used, especially that for which the difference in $V_{DS}$ between the diodes and M4 does not significantly affect $V_{TH}$, are not entirely met.

**Figure 7:**
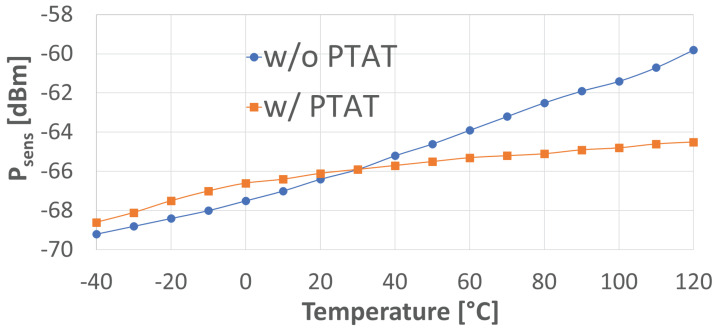
Version 1 simulated $P_{sens}$ with and without the use of the PTAT block.

According to [12], at the theoretical sensitivity, the SNR at the output of the amplifier is equal to $SNR_{req}=4.1$. With reference to Figure 3, the values for $V_{AV}$ corresponding to the theoretical sensitivity $V_{sens}$ are estimated through simulations. Figure 7 shows the values for $P_{RF,ant}$ corresponding to $V_{sens}$, i.e. $P_{sens}$, when the PTAT block is used and when it is not used. In both cases, NF of the subsequent amplifier is considered equal to unity, as mentioned above. The input matching network is a standard LC network with Q=80 [12], whereas $C_{in}$ is assumed equal to 3 pF, where $C_{in}^{\prime }=$ 1.68 pF and $C_{pad}$ is supposed to be close to 1.3 pF, which is plausible. ED noise bandwidth $f_{N}$ is around 1 kHz, i.e., around $f_{S}$. When the PTAT block is not used, $V_{B}$ and $V_{C}$ are set to the values corresponding to those set by the PTAT block at room temperature. Simulations were carried out from −40 °C to 120 °C and show that the use of the PTAT block has a positive impact on the stability of sensitivity over temperature, actually yielding a 5 dB reduction in sensitivity temperature variation across the operating range, as reported in Figure 7. As a matter of fact, the worst impact of the lack of compensation appears to be at high temperatures, most likely due to the fact that, here, $R_{in}$ without PTAT is lower than its nominal designed value, i.e., at 20 °C, drastically reducing $A_{v}$ as in (5). This also explains why sensitivity is affected very little at low temperatures.

### 4.4. Implementation Comparison

**Figure 8:**
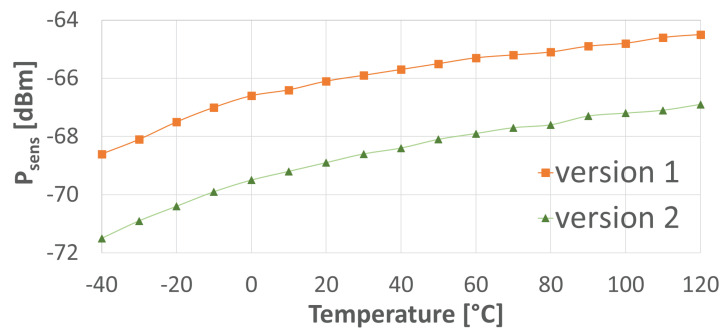
Simulated $P_{sens}$ of versions 1 and 2, both with the use of the PTAT block.

A comparison between the two implemented versions is carried out in terms of estimated overall chain sensitivity. Figure 8 reports the values for $P_{sens}$ with the use of the PTAT block employing versions 1 and 2, again assuming pad parasitics to be around 1.3 pF. The nominal sensitivity trend across temperature looks similar in the two cases, but sensitivity is better for version 2, i.e., with minimal diodes, as the corresponding curve is shifted downwards by an amount of 2–3 dBs. We carried out 100-run Monte Carlo simulations of versions 1 and 2 of the ED at 120 °C, which for both designs is the worst-case scenario in terms of sensitivity. They demonstrated a 1-σ sensitivity spread of 0.44 dB and 1.09 dB for versions 1 and 2, respectively, indicating the overall superior sensitivity performance of version 2.

Table 3 summarizes the main results for the two versions.

## 5. Conclusions

EDs are often used in communication applications, such as in Wake-Up Receivers, which are widely employed in IoT Wireless Sensor Networks. The target of this paper is to develop a passive ED with a temperature compensation mechanism in order to reduce the overall receiving chain sensitivity loss due to temperature changes. This is accomplished by keeping the input resistance of the ED constant with temperature with the aim of making it a constant load for the preceding matching network.

The central idea of the paper has been proven through a design in an STMicroelectronics 90 nm CMOS technology, receiving 1 kbps OOK-modulated packets with a 433 MHz carrier frequency and a 0.6 V supply. The use of the PTAT block yields a 5 dB reduction in sensitivity temperature variation across the −40 °C to 120 °C range. Moreover, two different implementations were compared, one targeting minimal mismatch and the other one targeting minimal area. The minimal area version appears to be better in terms of estimated overall chain sensitivity at all temperatures, despite a higher sensitivity spread.

## Figures and Tables

**Table 1:** Temperature stability comparison table with the state-of-the-art.

	This Paper	[15]	[17]	[18]	[19]
$P_{sens}$ at 20 °C [dBl	−66.1/−68.9	−76.3	−62.5	−72.4	−70.2
$\Delta P_{sens}$ for $T_{min}÷T_{max}$ [dB]	4.6	3.9	3	2.5	N/A
$T_{min}$ [°C]	−40	−10	−10	−30	−40
$T_{max}$ [°C]	120	50	40	70	85
FoM= $1/\left(\Delta P_{sens}/\left(T_{max}-T_{min}\right)\right)$ [°C/dB]	34.8	15.4	16.7	40	N/A

**Table 2:** Useful formulas for sensitivity optimization—Section 3.1.

ED input resistance	$R_{in}=r_{DS}/N$
ED input capacitance	$C_{in}=NC_{gs}+C_{pad}$
Matching network gain	$A_{v}=\sqrt{\frac{R_{in}}{R_{S}}}/\sqrt{\left(1+\frac{\omega R_{in}C_{in}}{Q_{ind}}\right)}$
Single-ended ED output voltage	$V_{ED}=\frac{NV_{M}^{2}}{4nV_{T}}$
ED delay	$\tau ∼CR_{in}\frac{N^{2}\left(N+1\right)}{2}$
Sensitivity	$P_{SEN}=\sqrt{\frac{SNR_{req}NF{\left(4nV_{T}\right)}^{2}k_{B}TR_{in}f_{S}}{A_{v}^{4}R_{S}^{2}}}$

**Table 3:** Comparison table between the two implemented versions.

	Version 1	Version 2
Target bitrate [kbit/s]	1	1
Power consumption at 20 °C [nW]	6	14
Diode dimensions [nm]	(450 × 2)/450	120/100
Coupling capacitances [fF]	36	13
$C_{in}$ [pF]	3	1.9
$R_{in}$ at 20 °C [kΩ]	10.7	10
$\Delta R_{in}$ for −40 ÷ 120 °C [kΩ]	24	13.7
$P_{sens}$ at 20 °C [dBm]	−66.1	−68.9
$\Delta P_{sens}$ for −40 ÷ 120 °C [dB]	4.6	4.6
$\sigma P_{sens}$ at 120 °C [dB]	0.4	1.1

## Data Availability

Data is contained within the article.

## Abbreviations

The following abbreviations are used in this manuscript:

ED	Envelope Detector
OOK	On–Off Keying
WuRX	Wake-Up Radio Receivers
IoT	Internet of Things
PTAT	Proportional-to-Absolute Temperature
MOSFET	Metal-Oxide-Semiconductor Field-Effect Transistor
SNR	Signal-to-Noise Ratio
NF	Noise Figure
